# Strengthen Village Malaria Reporting to Better Target Reservoirs of Persistent Infections in Southeast Asia

**DOI:** 10.1093/cid/ciy793

**Published:** 2018-10-22

**Authors:** Thomas J Peto, Rupam Tripura, Richard J Maude

**Affiliations:** 1 Mahidol Oxford Tropical Medicine Research Unit, Faculty of Tropical Medicine, Mahidol University, Bangkok, Thailand; 2 Nuffield Department of Clinical Medicine, University of Oxford, United Kingdom; 3 Department of Epidemiology, Harvard T. H. Chan School of Public Health, Harvard University, Boston, Massachusetts

**Keywords:** asymptomatic infection, community health worker, elimination, malaria, Southeast Asia

To the Editor—The recent World Health Organization malaria surveillance, monitoring, and evaluation manual highlights the importance that strengthened community health worker (CHW) programs and their ability to report accurate and timely data hold for the elimination of malaria [1]. Mass Drug Administration (MDA) is proposed as a means of interrupting *Plasmodium falciparum* transmission in areas of emergent, multidrug-resistant parasites [2, 3]. The 2017 World Health Organization recommendations on MDA inform control programs how to implement this strategy, but there is no specific advice on how to target suitable populations in Southeast Asia [4, 5].

Since 2013, we have conducted population-based surveys to define the micro-epidemiology of asymptomatic malaria infections and have piloted MDA in Southeast Asia [6]. Asymptomatic *P. falciparum* infections persist, on average, for several months, with varying parasite densities that are periodically capable of transmission [7]. Our experience is that prevalence surveys are an expensive and time-consuming means of identifying foci of transmission in pre-elimination (low-transmission) settings, particularly where highly-sensitive molecular techniques are used to detect asymptomatic infections. Currently, CHWs are active in many more villages than could be practicably included in a baseline prevalence survey, but are well positioned—with strengthening of the reporting system where needed—to routinely collect travel and residency data to determine whether individual locations are sources where transmission occurs or sinks where cases are reported but not acquired.

If of sufficient quality, CHW data could be used to identify locations for targeted MDA, such as village clusters where the *P. falciparum* incidence is above a locally-defined threshold. High-quality incidence data has been shown to be predictive of asymptomatic carriage rates in low-transmission settings, thus potentially obviating the need to screen populations using more expensive molecular methods to define targets for MDA [8]. Incidence data determined from reliable case reporting could also be the preferred metric to evaluate the impact of MDA. For example, a recent elimination program in Myanmar demonstrated a rapid decline in the incidence of malaria following the implementation of a strong village malaria worker network, demonstrating the effectiveness of conducting an MDA in a transmission hotspot [9].

In Southeast Asia, asymptomatic *Plasmodium vivax* infections are even more under-detected and undertreated than *P. falciparum* [10]. In our studies, a history of clinical malaria was a consistently strong risk factor for persistent asymptomatic infection. In a prior survey, we matched participants to treatment records and found that approximately a third of people with a history of clinical *P. vivax* were parasitaemic [11]. Therefore, local health services already have recorded the names and locations of thousands of people harboring *P. vivax* infections that contribute to ongoing transmission. These people could be screened for G6PD deficiencies and offered safe treatment with primaquine for radical cures of liver-stage parasites. Targeting persistent *P. vivax* from treatment records alone would neither catch all carriers nor interrupt transmission, but could treat an important fraction of extant *P. vivax* infections and represent a move from *P. vivax* control towards elimination.

As countries progress towards elimination, investments in strengthening and expanding the coverage of CHW programs and case reporting are vital. Making better use of this data could identify persistent infections at both the community and individual levels, allowing for the targeting of elimination strategies that address the asymptomatic reservoir and for new screen-and-treat strategies, which may become viable with the deployment of highly-sensitive rapid diagnostics.

## Note

***Potential conflicts of interest.*** All authors: No reported conflicts of interest. All authors have submitted the ICMJE Form for Disclosure of Potential Conflicts of Interest. Conflicts that the editors consider relevant to the content of the manuscript have been disclosed.
